# Perception of urge-to-cough and dyspnea in healthy smokers with decreased cough reflex sensitivity

**DOI:** 10.1186/1745-9974-6-1

**Published:** 2010-02-05

**Authors:** Masashi Kanezaki, Satoru Ebihara, Etsuhiro Nikkuni, Peijun Gui, Chihiro Suda, Takae Ebihara, Miyako Yamasaki, Masahiro Kohzuki

**Affiliations:** 1 Department of Internal Medicine and Rehabilitation Science, Tohoku University Graduate School of Medicine, Seiryo-machi 1-1, Aoba-ku, Sendai 980-8574, Japan; 2 Department of Geriatrics and Gerontology, Institute of Development, Aging and Cancer, Tohoku University, Seiryo-machi 4-1, Aoba-ku, Sendai 980-8575, Japan

## Abstract

**Background:**

Although cigarette smoking has been implicated as an important risk factor for the development of respiratory symptoms, the perceptional aspects of two symptoms in smokers have not been fully elucidated. Therefore, we simultaneously evaluated the cough reflex sensitivity, the cognition of urge-to-cough and perception of dyspnea in both healthy smokers and non-smokers.

**Methods:**

Fourteen male healthy never-smokers and 14 age-matched male healthy current-smokers were recruited via public postings. The cough reflex sensitivity and the urge-to-cough were evaluated by the inhalation of citric acid. The perception of dyspnea was evaluated by Borg scores during applications of external inspiratory resistive loads.

**Results:**

The cough reflex threshold to citric acid, as expressed by the lowest concentration of citric acid that elicited two or more coughs (C_2_) and the lowest concentration of citric acid that elicited five or more coughs (C_5_) in smokers was significantly higher than in non-smokers. The urge-to-cough log-log slope in smokers was significantly milder than that of non-smokers. There were no significant differences in the urge-to-cough threshold between non-smokers and smokers. There were no significant differences in perceptions of dyspnea between non-smokers and smokers.

**Conclusions:**

The study showed that decreased cough reflex sensitivity in healthy smokers was accompanied by a decreased cognition of urge-to-cough whereas it was not accompanied by the alternation of perception of dyspnea. Physicians should pay attention to the perceptual alterations of cough in smokers.

## Background

Cough and dyspnea are common respiratory symptoms for which patients seek medical attention. Although cigarette smoking has been implicated as an important risk factor for the development of respiratory symptoms [1-3], the perceptional aspects of cough and dyspnea in smokers have not been fully elucidated. Since tobacco smoking is also associated with an increase in respiratory and non-respiratory infections [4], it is of importance in a clinical setting to know whether perceptional alternations of these two symptoms occur in smokers, and if so, how they are related. However, there have been few studies which investigated both the perceptions of cough stimuli and dyspneic stimuli in smokers.

Although dyspnea is a respiratory sensation, cough is a motor action typically preceded by a respiratory sensation such as an awareness of an irritating stimulus and is perceived as a need to cough, termed the urge-to-cough [5]. Urge-to-cough is a component of the brain motivation system that mediates the cognitive responses of cough stimuli [6]. Cough reflex sensitivity is severely diminished during general anesthesia or sleep [7,8]. In patients with congenital central hypoventilation syndrome and aspiration pneumonia, both the cough reflex sensitivity and the cognition of cough are significantly impaired [9,10]. These studies suggest that the initiation of a cough reflex response is facilitated by the cognition of the urge-to-cough.

Both the urge-to-cough and dyspnea are uncomfortable respiratory sensations. The perceptions of the urge-to-cough and dyspnea may share common pathways and somatosensory areas [11]. Both the urge-to-cough and dyspnea can arise from stimulation by chemical substances and changes in the mechanical environment acting on receptors in the lung and airways [12]. Some pulmonary and airway sensory receptors and afferent pathways may be common to both the urge-to-cough and dyspnea [11]. In addition, brain imaging studies showed the brain cortical areas related to the urge-to-cough and dyspnea overlap [13-15]. Therefore, if the common sensory afferent pathways and/or cortical areas are involved in cough reflex sensitivity which is known to be modulated by tobacco smoking, the perceptions of the urge-to-cough and dyspnea might be changed simultaneously. However, no study has investigated the perception of dyspnea together with cognition of the urge-to-cough in smokers.

Therefore, in the present study, we investigated the cough reflex sensitivity, the cognition of the urge-to-cough and the perception of dyspnea simultaneously in healthy male smokers using citric acid as a tussive stimuli and external inspiratory resistive load as a dyspnea intervention.

## Methods

### Subjects

Fourteen male healthy never-smokers and 14 male healthy current-smokers were allocated to evaluate cough related responses to inhaled citric acid and dyspnea sensation during inspiratory resistive loads. All were originally recruited via public postings in and around the Tohoku University School of Medicine campus. The mean age was 30.0 ± 4.9 (SD) years. The study was approved by the Institutional Review Board of the Tohoku University School of Medicine. Subjects were without history of pulmonary and airway diseases, recent (within 4 weeks) suggestive symptoms, respiratory tract infection, and seasonal allergies. Subjects did not take any regular medication.

### Cough reflex sensitivity and urge-to-cough

Cough reflex, the urge-to-cough, the perception of dyspnea and spirometry were examined at around 2:00 pm for each subject. The smokers smoked more than one cigarette within 2 hours of evaluation. Simple standard instructions were given to each subject.

Cough reflex sensitivity to citric acid was evaluated with a tidal breathing nebulized solution delivered by an ultrasonic nebulizer (MU-32, Sharp Co. Ltd., Osaka, Japan) [10,16]. The nebulizer generated particles with a mean mass median diameter of 5.4 μm at an output of 2.2 ml/min. Citric acid was dissolved in saline, providing a two-fold incremental concentration from 0.7 to 360 mg/ml. The duration of each citric acid inhalation was 1 minute. Based on the "cough sound", the number of coughs was counted both audibly and visually by laboratory technicians who were unaware of the clinical details of the patients and the study purpose. Each subject inhaled a control solution of physiological saline followed by a progressively increasing concentration of citric acid. Increasing concentrations were inhaled until five or more coughs were elicited, and each nebulizer application was separated by a 2 minute interval. The cough reflex sensitivities were estimated by both the lowest concentration of citric acid that elicited two or more coughs (C_2_) and the lowest concentration of citric acid that elicited five or more coughs (C_5_) during 1 minute.

Immediately after the completion of each nebulizer application, the subject made an estimate of the urge-to-cough. The modified Borg scale was used to allow subjects to estimate the urge-to-cough [5]. The scale ranged from "no need to cough" (rated 0) and "maximum urge-to-cough" (rated 10). The urge-to-cough scale was placed in front of the subjects and the subject pointed at the scale number, which was recorded by the experimenter. To assess the intensity of the urge-to-cough, subjects were recommended to ignore other sensations such as dyspnea, burning, irritation, choking, and smoke in their throat. Subjects were told that their sensation of an urge-to-cough could increase, decrease, or stay the same during the citric acid challenges, and that their use of the modified Borg scale should reflect this.

In each subject, the estimated urge-to-cough scores were plotted against the corresponding citric acid concentration using a log-log transformation. Since it is known that there is a linear relationship between estimated urge-to-cough scores and tussive agent concentration on a log-log scale [5,17], the slope and intersection were determined by linear regression analysis on a log-log scale. The thresholds of the urge-to-cough in each subject were estimated as an intersection with the X-axis (citric acid concentration axis), indicating the dose of the urge-to-cough score = 1.

### Perception of dyspnea

Dyspnea was induced by introducing an inspiratory resistive load to the external breathing circuit and was assessed by the modified Borg scale [18,19]. In brief, the sensation of dyspnea was measured while the subject breathed through the Hans-Rudolph valve with a linear inspiratory resistance (R) of 10, 20, and 30 cmH_2_O/L/s. The loads were presented with increasing magnitudes. Neither ventilation nor breathing pattern was controlled during the test. After breathing for 1 minute at each level of resistance, the subject rated the sensation of dyspnea [discomfort of breathing] using the modified Borg scale. This is a category scale in which the subject selects a number, from 0 (no dyspnea) to 10 (maximal dyspnea), describing the magnitude of the sensation of dyspnea. At the beginning of the measurement each subject was asked to rate the sensation of "kokyu-konnan" or "discomfort of breathing" while breathing with resistances. The term "kokyu-konnan" is an exact Japanese translation of "dyspnea" ("kokyu" means breathing or respiration and "konnan" means discomfort or difficulty). In Japan this is not a special term, and most people understand the meaning of it. The term "kokyu-konnan", or discomfort of breathing was not defined any further, but the subjects were instructed to avoid rating non-respiratory sensations such as headache or irritation of the pharynx.

In order to exclude the mouth piece effect the perception of dyspnea in individuals, the scores at each resistive load were subtracted by the score at R = 0 cmH_2_O/L/s. After subtractions, comparisons were performed in the Borg score at each load, and summation of the Borg scores of the 3 loads applied. Since it is known that there is a linear relationship between amount of load and Borg dyspnea scores [20,21], we also estimated the linear regression slope with least square fitting when estimated Borg scores were plotted against the corresponding amounts of resistive loads.

### Data analysis

The study protocol was approved by the local ethics committee and informed consent was obtained from all subjects. Data are expressed as mean (SD) except where specified otherwise. The Mann-Whitney *U *test was used to compare patients with controls. A p value of < 0.05 was considered significant.

## Results

All 28 men completed the experiments without any difficulty or side effects. The characteristics of subjects are summarized in Table 1. There was no significant difference in age, height, body weight, and spirometry data between the non-smokers and smokers. The smokers smoked 12.4 ± 5.7 cigarettes/day for 8.6 ± 4.9 years.

**Table 1:** Comparison of characteristics between non-smokers and smokers

	Non-smokers	Smokers	P- value
Number	14	14	
Age (years)	30.4 ± 3.4	29.6 ± 4.5	n.s.
Height (cm)	173.8 ± 3.5	172.7 ± 4.7	n.s.
Weight (kg)	69.2 ± 13.8	65.9 ± 9.2	n.s.
Pack-years	0 ± 0	5.6 ± 4.9	
FEV_1 _(L)	4.16 ± 0.54	4.03 ± 0.46	n.s.
FEV_1 _(% predict)	104.5 ± 11.6	101.9 ± 13.0	n.s.
FVC (L)	4.86 ± 0.63	4.64 ± 0.55	n.s.
FVC (% predict)	107.8 ± 30.7	115.2 ± 13.3	n.s.
FEV_1_/FVC (%)	85.8 ± 4.6	86.9 ± 3.6	n.s.

Data are mean ± S.D. P-values were calculated by the Mann-Whitney *U *test. n.s. denotes not significant.

**Figure 1:**
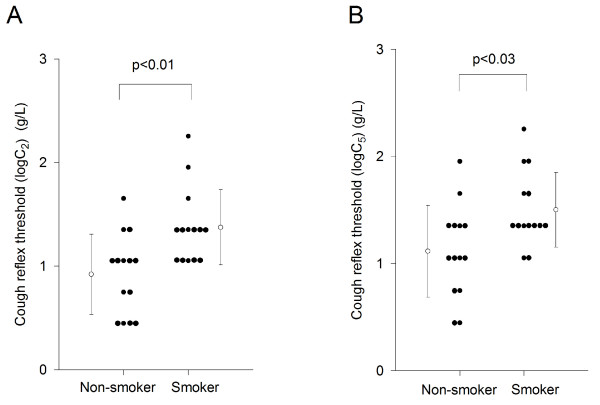
**Comparisons of cough reflex sensitivity between non-smokers and smokers**. (A) Cough reflex sensitivities are expressed as the log transformation of the lowest concentration of citric acid that elicited two or more coughs (C_2_). (B) Cough reflex sensitivities are expressed as the log transformation of the lowest concentration of citric acid that elicited five or more coughs (C_5_). Open circles and error bars indicate the mean value and the standard deviation in each group, respectively.

As shown in Figure 1A, the cough reflex threshold to citric acid, as expressed by log C_2_, in smokers (1.37 ± 0.36 g/L) was significantly higher than that of non-smokers (0.92 ± 0.39 g/L, p < 0.01). Similarly, the cough reflex threshold to citric acid, as expressed by log C_5_, in smokers (1.50 ± 0.35 g/L) was significantly higher than that of non-smokers (1.12 ± 0.43 g/L, p < 0.05) (Figure 1B).

**Figure 2:**
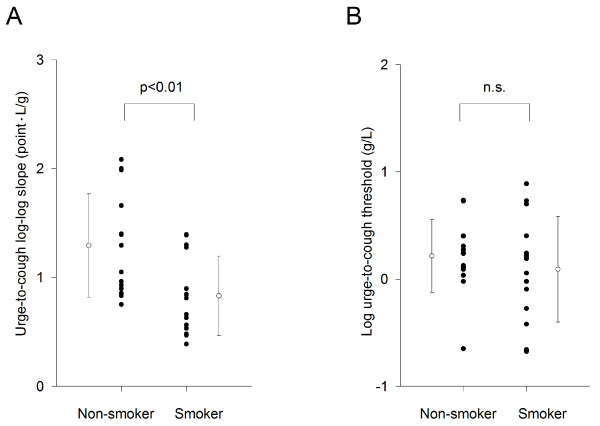
**Comparisons of and the urge-to-cough between non-smokers and smokers**. (A) The urge-to-cough log-log slope by linear regression between log citric acid concentration and the log Borg scores. (B) The urge-to-cough threshold estimated by log citric acid concentration at the log Borg Score of urge-to-cough = 0. Closed circles indicate the value of each subject. Open circles and error bars indicate the mean value and the standard deviation in each group, respectively. n.s. denotes not significant.

The log-log slope between citric acid concentration and the Borg scores of the urge-to-cough was estimated for each subject. The urge-to-cough log-log slope in smokers (0.83 ± 0.36 points • L/g) was significantly milder than those of non-smokers (1.29 ± 0.47 points • L/g, p < 0.01) (Figure 2A). The urge thresholds were estimated as the intersection with the X-axis (log citric acid concentration) of the linear regression equation of the log-log relationships between citric acid concentration and the Borg scores of the urge-to-cough There were no significant differences in the urge-to-cough threshold estimated between non-smokers (0.22 ± 0.34 g/L) and smokers (0.09 ± 0.49 g/L) (Figure 2B).

Table 2 shows the perception of dyspnea during the external inspiratory resistive loads. There were no significant differences between non-smokers and smokers in the Borg scores at each load and at summation. When the slope of the Borg score change was estimated as a function of the amount of loads by linear regression in each subject, there was no significant difference between non-smokers and smokers

**Table 2:** Comparison of perceptions of dyspnea between non-smokers and smokers

	Non-smokers	Smokers	P- value
Number	14	14	
R = 10 (point)	2.3 ± 1.0	1.9 ± 1.3	n.s.
R = 20 (point)	3.1 ± 1.4	2.9 ± 1.5	n.s.
R = 30 (point)	4.4 ± 1.5	4.8 ± 1.8	n.s.
Sum (point)	9.7 ± 3.8	9.8 ± 4.8	n.s.
Slope (point • L/g)	0.14 ± 0.05	0.15 ± 0.05	n.s

Data are mean ± S.D. R = 10, R = 20 and R = 30 indicates the Borg score at R = 10, R = 20 and R = 30 cmH_2_O/L/s, respectively. Sum indicates the summation of Borg scores at R = 10, R = 20 and R = 30 cmH_2_O/L/s. Slope indicates the linear regression slope when estimated Borg scores were plotted against the corresponding values of resistive loads. P-values were calculated by the Mann-Whitney *U *test. n.s. denotes not significant.

## Discussion

In this study, healthy smokers showed a depressed cough reflex sensitivity accompanied by a depressed cognition of the urge-to-cough whereas the perception of dyspnea during external inspiratory resistive loading did not significantly alter.

Both enhanced and diminished cough sensitivities to tussive agents have been reported in chronic smokers [22-26]. The wide range of differences in smoking pattern and history and existing airway dysfunction, were probably related to the balance between up-regulating and down-regulating factors of cough reflex sensitivity. The mechanism of up-regulation of cough reflex sensitivity by tobacco smoking is well characterized in animal studies which consistently show that chronic exposure to cigarette smoke induces enhanced cough responses to various inhaled tussive agents [27-29]. However, the underlying mechanisms for the down-regulation of cough reflex sensitivity in smokers are not fully understood.

Although cough is usually referred to as a reflex controlled from the brainstem, cough can be also controlled via the higher cortical center and be related to cortical modulations [30]. Therefore, the depression of cough reflex could be due to the disruption of both the cortical facilitatory pathway for cough and the medullary reflex pathway. Since the urge-to-cough is a brain component of the cough motivation-to-action system, the depressed urge-to-cough suggests impairment of supramedullary pathways of cough reflex [6].

It is reasonable to suppose that urge-to-cough arises from sensors that mediate cough reflex. In the bronchopulmonary system, there are at least five sensors involved in this reflex [12]. The dyspnea sensation induced by external resistive loads is reported to be described as the work/effort sensation of dyspnea [31-33]. The neural pathways proposed for this sensation include corollary discharge from motor cortical centers that drive voluntary breathing, and muscle mechanoreceptors and metaboreceptors [33]. Although tobacco smoke may induce desensitization of bronchopulmonary sensors or structural changes interfering with accessibility to sensors [34,35], it is less possible to affect muscle mechanoreceptors and metaboreceptors in healthy young smokers. Therefore, the differential susceptibility to tobacco smoke in peripheral receptors/sensors may explain the dissociation of perceptions of the urge-to-cough by citric acid and dyspnea during external resistive loads. However, in the present study, although cough reflex sensitivity and the urge-to-cough log-log slope were decreased in smokers, the urge-to-cough thresholds did not change (Figure 2). This may suggest no significant changes in bronchopulmonary sensors involved in the urge-to-cough induction and the larger contribution of central gain mechanisms rather than the peripheral ones.

Davenport et al. showed that nicotine administration inhibited urge-to-cough rating scores in smokers deprived from smoking for more than 12 hours [36]. In this study, smokers who withdrew from tobacco smoke showed a greater number of coughs, higher urge-to-cough rating and higher anxiety scores than non-smokers, and the nicotine administration reduced those to match the non-smokers. The study clearly showed the role of nicotine on the central modulation of cough cognitive motivational system and motor response. However, due to a lack of the data concerning smokers without withdrawal from tobacco smoke, the state of cough cognitive motivational system in smokers with depressed cough reflex sensitivity has not been elucidated.

In the present study, we showed the cough cognitive motivational system was inhibited in smokers with depressed cough reflex sensitivity. Since it was reported that nicotine and tobacco smoking induce the endogenous opioid system [37], cognition of the urge-to-cough might be inhibited by endogenous opioids in smokers. However, this is unlikely because we failed to detect the depressed perception of dyspnea which is also inhibited by endogenous opioids [38]. To our knowledge, the depressed perception of dyspnea has not been reported in healthy smokers.

Respiratory sensation such as various types of dyspnea and the urge-to-cough are the result of sensory activation of subcortical and cortical neural pathways. Some of these pathways are shared across respiratory modalities while activation of some neural areas are modality specific [15]. There are many brain imaging studies concerning dyspnea using different techniques to induce dyspnea. Despite the use of different intervention techniques, the common predominant neural activity has been found in the insula, operculum, and frontal cortex areas, the anterior cingulated cortex, the posterior cingulated cortex, the cerebellum, the thalamus, and the amygdala [13,39]. In contrast, there is only one brain imaging study concerning the urge-to-cough by Mazonne et al. [14]. Their functional magnetic resonance imaging study showed activation in insula, anterior cingulated, primary sensory cortex, orbitofrontal cortex, supplementary motor area and cerebellum during the induction of the urge-to-cough by capsaicin [14]. Although it is still unclear how these brain regions relate to the respiratory sensations, our study may suggest that shared brain regions, such as insula, anterior cingulated, and cerebellum, which are activated by both dyspnea and urge-to-cough are not suppressed by tobacco smoke. Since it has been proposed that initiation of a reflex cough response requires the urge-to-cough to facilitate it [6], the depressed cough reflex sensitivity in healthy smokers might be explained solely by the supramedually mechanism.

Cigarette smoking appears to be a major risk factor for respiratory tract infections [4]. As cough is a normal reflex and respiratory defense mechanism, blunted cough reflex sensitivity may contribute to the risk of respiratory tract infection in cigarette smokers. Moreover, since dyspnea is usually a symptom at a relatively advanced stage of respiratory tract infection whereas cough represents at earlier stages, the blunted urge-to-cough may contribute to the development of respiratory tract infections in smokers due to failure to seek proper medical service.

## Conclusions

Our study showed that decreased cough reflex sensitivity in healthy smokers was accompanied by a decreased cognition of the urge-to-cough whereas it was not accompanied by the alternation of perception of dyspnea. Physicians should pay attention to the perceptual alterations of cough in smokers.

## Abbreviations

C_2_: the lowest concentration of citric acid that elicited two or more coughs; C_5_: the lowest concentration of citric acid that elicited five or more coughs.

## Competing interests

The authors declare that they have no competing interests.

## Authors' contributions

MK and SE participated in the design of the study, collected and analyzed data, and drafted the manuscript. EN, PG, CS and MY participated in the design of the study and collected the data. TE and MK participated in design of the study and helped to draft the manuscript. All the authors read and approved the final manuscript.
